# Epidemiological study of soft‐tissue sarcomas in Ireland

**DOI:** 10.1002/cam4.547

**Published:** 2015-11-21

**Authors:** Nikita Bhatt, Sandra Deady, Amy Gillis, Alexia Bertuzzi, Aurelie Fabre, Eric Heffernan, Charles Gillham, Gary O'Toole, Paul F. Ridgway

**Affiliations:** ^1^ Department of SurgeryAdelaide and Meath HospitalTallaghtDublinIreland; ^2^ National Cancer Registry IrelandCorkIreland; ^3^ Department of Medical OncologyAdelaide and Meath HospitalTallaghtDublinIreland; ^4^ Department of HistopathologySt. Vincent's University HospitalDublinIreland; ^5^ Department of RadiologySt. Vincent's University HospitalDublinIreland; ^6^ Department of Radiation OncologySt Luke's HospitalDublinIreland; ^7^ Department of OrthopaedicsSt. Vincent's University HospitalDublinIreland

**Keywords:** Incidence, rare cancers, soft‐tissue sarcomas, survival

## Abstract

Soft‐tissue sarcomas (STS) account for 1% of adult and 7% of pediatric malignancies. Histopathology and classification of these rare tumors requires further refinements. The aim of this paper is to describe the current incidence and survival of STS from 1994 to 2012 in Ireland and compare these with comparably coded international published reports. This is a retrospective, population study based on the data from the National Cancer Registry of Ireland (NCRI). Incidence and relative survival rates for STS in Ireland were generated. Incidence of STS based on gender, age and anatomical location was examined. Annual mean incidence rate (European Age Standardized) in Ireland between 1994 and 2012 was 4.48 ± 0.15 per 100,000 person‐years. The overall relative 5‐year survival rate of STS for the period 1994–2011 in Ireland was 56%, which was similar to that reported in the U.K. but lower than in most of Europe and U.S.A. Survival rate fluctuated over the period examined, declining slightly in females but showing an increase in males. STS incidence trends in Ireland were comparable to international reports. Survival trends of STS were significantly different between Ireland and other European countries, requiring further study to understand causation.

## Introduction

Soft‐tissue sarcomas (STS) account for 1% 1, 2 of adult and 7% of pediatric malignancies 3. These heterogeneous mesenchymal neoplasms can arise from any anatomical site. They occasionally may be life threatening at presentation but usually present in a sub acute fashion. More than 50 different subtypes of STS each having unique clinical, prognostic and therapeutic characteristics, pose as a diagnostic and therapeutic challenge 1, 4.

To our knowledge, there are no peer reviewed publications describing the incidence and survival of patients diagnosed with STS in Ireland. The Dublin Soft Tissue Tumour Panel established in 1989 reported the prevalence and management of STS in four major centers of Dublin in the 90s 5, 6. Part based on the work for this study, a trends report on STS was published recently by the National Cancer Registry of Ireland (NCRI) on its website 7.

The classification and characterization of these tumors continue to evolve over time as immunohistochemical and molecular analysis and an improved understanding of the underlying genetic changes have supplemented the knowledge on their histopathology. But, despite significant progress on our understanding of STS from a histopathologic and genetic point of view, more refinement is needed until we have universally applicable coding practices. This remains one of the most significant challenges faced by diagnostic and treating physicians today.

Different reporting authorities around the world do not follow a standard grouping method for STS. For example in the Standard Surveillance, Epidemiology and End Results (SEER) program tabulation from the United States, STS only include sarcomas arising from the soft tissue, but not that arising in the specific organs such as skin and organ sites. So, the SEER tabulations grossly underestimate the true STS rates in the United States. 2. STS are identified by a combination of their ICD‐O site and morphology codes, the United Kingdom identifying all STS as those sarcomas sited outside of bone (ICD10: C40‐C41) and brain and central nervous system (ICD10: C70‐C72) 8. In an effort to standardize STS reporting for European countries, RARECARE developed a 3 tier coding system. The tier 3 in this classification corresponds to the WHO ICD‐O. Tier 3 entities have been grouped into tier 1 and 2 entities, which, on a consensus basis, were felt to be homogeneous form the clinical standpoint. Tier 2 is defined by the anatomical primary tumor site 4. Hence, different reporting authorities use different methods for classifying STS. In addition, the coding practices have changed significantly over time, gastrointestinal stromal tumors (GIST) were recognized as a separate entity only after gaining its own code in 2000 (ICD‐03). This introduces an unavoidable bias when looking at STS trends or using data over a long time period as RARECARE exclude GIST from their overall STS grouping.

Despite advances in the knowledge of the molecular genotypes of STS, the etiology of these tumors is still not fully understood. Few predisposing or associated factors have been identified. Exposure to ionizing radiation especially during cancer treatments is one of the external factors responsible for development of a STS. The prevalence of STSs is higher in patients with certain inherited syndromes like Li‐Fraumeni mutation (p53 mutation), Gardner syndrome (*APC* mutation), von Recklinghausen disease (neurofibromatosis type 1: *NF1* mutation) etc. 8, 9, 10. Wider geographical population based etiological factors remain unknown.

The aim of this paper was to describe the trends in the incidence and survival of STS from 1994 to 2012 in Ireland. We compared the data with relevant published international reports that utilized similar coding methodology. We also reported incidence rates of STS with respect to age, gender and anatomical location.

## Methods

### Data collection

This is a retrospective, population based analysis of the incidence and survival of STS in Ireland. A literature review on the incidence and survival, gender and age distribution of STS as well as comparison of data from different countries was done. The study is based on data from the NCRI.

Patients with STS were identified using data from the NCRI in the period between 1994 and 2012. The data collected includes the demographic characteristics of the patients (age, sex and county of residence), site of tumor according to the International Classification of Diseases for Oncology, third revision (ICD‐O‐3) and the year of diagnosis. All the cases that met the morphologic criteria of STS as per the grouping by RARECARE, Europe were included in the study 11.

NCRI has been collecting data on all cancer cases diagnosed in Ireland since 1994 and it covers a current population of 4.5 million 12. The data sources for this registry include hospital inpatient records, General Practitioner Notifications, Nursing home and district hospital data, National death certificate data, screening pathology and medical cards as well as prescription data 13.

We obtained the annual average number of new cases of STS diagnosed in Ireland between 1994 and 2012. Age standardized rates (ASR) per 100,000 person‐years in Ireland were calculated, using the European standard population. The trends in ASR over time were examined using regression analysis.

The 5‐year relative survival rates of patients with STS from 1994 until 2011 in Ireland were reported. The rates were reported for cases diagnosed up to the end of 2011 to allow minimum follow up of 1 year for all cases. The average 5‐year survival rates were then compared to International Reports from the United States and Europe.

The Age Specific Rates at 5‐year intervals from 0 to >85 years were examined. The incidence rates were described with respect to gender. The total number of new cases at each anatomical site was reported for Ireland and the ASR in Ireland compared to international reports from the United States and Europe.

### Statistical analysis

ASRs were weighed against the European standard population. Annual percentage change of incidence over time was calculated using the Joint point regression program 14. Relative survival was calculated using the “strs” command in STATA 11 (StataCorp LP, TX).

## Results

### Age‐adjusted incidence rate

The annual average case number of STS in Ireland over the period from 1994 to 2012 was 176. The annual mean age‐adjusted incidence rate (ASR) in Ireland was 4.48 ± 0.15 per 100,000 person‐years from 1994 to 2012. The total number of cases of STS in Ireland during this period was 3339.

**Figure 1:**
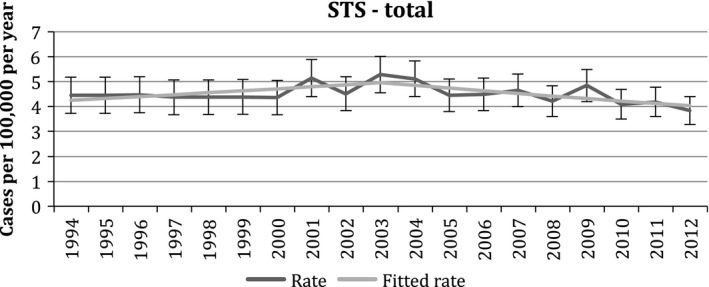
Trends in age‐adjusted incidence rates of soft‐tissue sarcomas (STS) in Ireland from 1994 to 2012.

The annual ASR ranged from 3.83 to 5.27 per 100,000 persons in Ireland for the period 1994 to 2012. The incidence of STS in Ireland over the period between 1994 and 2012 has fluctuated over time. Incidence rates of STS increased from 1994 to 2003 by an annual percentage of 1.71 (95% CI: −0.09, 3.54), while the rates subsequently declined significantly by an annual percentage of −2.64 (95% CI: −4.77, −0.65) (Fig. 1). Although not included in the overall STS grouping as defined by RARECARE, and so excluded from this analysis, an annual average of 20 GIST were also diagnosed per year since 2005 (when ICD‐O3 coding was adopted by the NCRI), representing an annual average ASR of 0.45 per 100,000 person‐years.

### Distribution of tumors by anatomical site between 1994 and 2012

The most frequent site for development of a STS in Ireland was the limbs (20%) followed by the uterus (16%) and skin (10%). STS of the limbs formed 83.8% of all invasive cancers at this site (excluding bone). A detailed breakdown of incidence according to tumor site is given in Table 1.

**Table 1:** Distribution of STS in Ireland according to the anatomical site

Location	Incidence rate (cases per 100,000 per year)		
	Females	Males	Total
STS of head and neck	0.16	0.35	0.24
STS of limbs	0.75	**1.09**	**0.90**
STS of superficial trunk	0.33	0.53	0.42
STS of mediastinum	0.01	0.01	0.01
STS of heart	0.02	0.02	0.02
STS of breast	0.38	0.00	0.19
STS of uterus	**1.44**	–	–
STS of paratestis	–	0.03	–
STSs of other genitourinary^a^	0.46	0.13	0.30
STS of viscera	0.23	0.31	0.27
STS of retroperitoneum and peritoneum	0.13	0.18	0.15
STS of pelvis	0.20	0.24	0.22
STS of skin	0.31	0.55	0.42
STS of paraorbit	0.01	0.01	0.01
STS of brain and other nervous system	0.15	0.16	0.15
Embryonal rhabdomyosarcoma of soft tissue	0.08	0.11	0.10
Alveolar rhabdomyosarcoma of soft tissue	0.04	0.06	0.05
Ewing's sarcoma of soft tissue	0.02	0.05	0.03
STS other and unspecified	0.20	0.27	0.23
Total STSs^b^	4.92	4.10	4.48

STS, soft‐tissue sarcoma; ASR, age standardized rates; GIST, gastrointestinal stromal tumors. Figures in bold indicate sites with highest ASR.

a Vulva, vagina, ovary, penis, prostate, testis, kidney, renal pelvis, ureter, bladder, and urethra.

b Excludes GIST and Kaposi sarcoma.

### Survival rates

**Figure 2:**
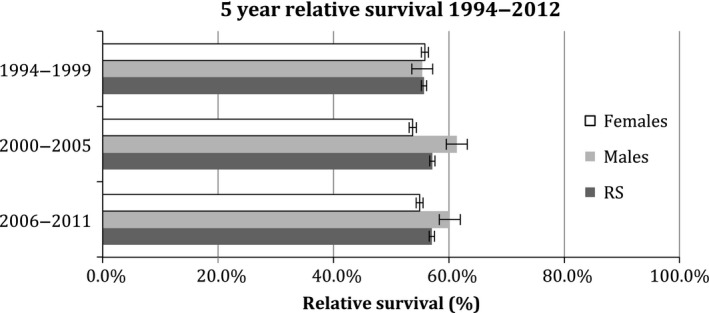
Trends in 5‐year survival rates of soft‐tissue sarcomas in Ireland from 1994 to 2011.

The 5‐year relative survival rate of STS in Ireland between 1994 and 2011 was 56.5% (95% CI 54.4–58.5). Five year relative survival for males was higher than for females (males: 58.9%, 95% CI 55.7–62.1; females: 54.7%, 95% CI 52.0–57.3). Survival fluctuated over the 18‐year period but there was little overall change in females and a slight increase in males (Fig. 2).

**Figure 3:**
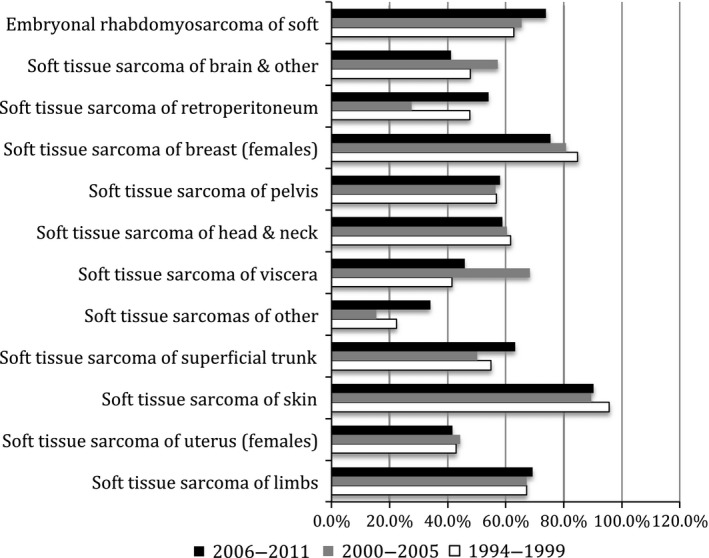
Five‐year relative survival rates according to site.

The 5‐year relative survival rate over 2006 to 2011 was highest for STS of the skin (91.0%) and lowest for the genitourinary tumors in females (uterus: 43.2% and others: 23.4%) (Fig. 3).

Tumour, Node, Metastasis (TNM) tumor staging does not apply to most STS histological subtypes and only 14% of all STS were assigned a tumor stage. The majority of cases where stage was known were stage IV.

### Comparison of incidence and survival trends of STS with published international reports

The incidence of STS in Ireland was similar to the United Kingdom (4.5 per 100,000 person‐years) 8 and Central as well as Southern Europe (4.5 per 100,000 person‐years) 9; but it was lesser than Northern Europe (4.7 per 100,000 person‐years) 9 and the United States (5.03 per 100,000 person‐years) 2. It was greater than Eastern Europe (3.3 per 100,000 person‐years) 2, 4, 15.

The overall 5‐year relative survival rates over the period from 1994 to 2011 in Ireland were similar to those in the United Kingdom (56%, 1996–2010) and close to European averages (57.8%, 1995–2002) but lower than in United States (65.1%, 1975–2011) and certain areas in Europe where survival was up to 61% 4, 15, 16.

### Age‐specific rates

**Figure 4:**
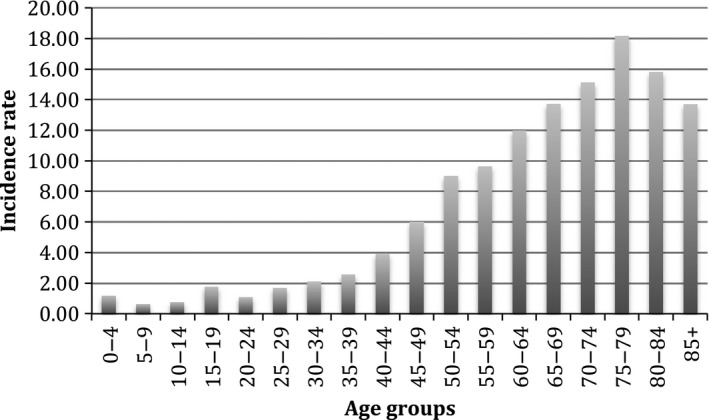
Age‐specific rates of soft‐tissue sarcomas per 100,000 person‐years in Ireland between 1994 and 2012.

Peak incidence of STS in the Irish population was found in the age group of 75–79 years (Fig. 4). Embryonal and alveolar rhabdomyosarcoma of the soft tissue were most commonly found in the pediatric population (0–14 years) while STS of the limbs and uterus were most commonly found in the older age groups (>60).

### Gender distribution

Both the number of cases of STS (M:F = 0.77:1) and the age‐adjusted incidence rates of STS (M:F = 0.837:1) were higher in females than in males in Ireland between 1994 and 2012. STS in females was most commonly found in the uterus (ASR: 1.44) and in males in the limbs (ASR: 1.09) (Table 1).

## Discussion

The age‐adjusted incidence rate of STS in Ireland was 4.48 per 100,000 person years. This incidence rate falls at the high end of the range of published international incidence rate (1.8 and 5.0 per 100,000 per year). The ASR in Ireland is similar to the United Kingdom but differs from other countries of central Europe and the United States 2, 4, 15, 17. The inclusion criteria, number of cases, classification and typing standards of STS may differ for each country and over various time periods. For example, a study from Sweden only included patients of age >16 years and STS of the extremities and trunk wall. This could be the explanation for the lower incidence rates seen in this country. All the other studies included patients irrespective of their age and all STS cases irrespective of the site 18. The 2006 U.S.A. study by Toro et al. sought to modify the “Standard Surveillance, Epidemiology and End Results” (SEER) database for analysis. SEER only included sarcomas arising in soft tissue, but not those arising in specific organs such as the skin and organ sites as Toro's group used in their report 2.

The difference in incidence could also be due to different population characteristics, geographical and topographical differences and influence of risk factors as well as the relatively low number of tumors diagnosed in the much smaller population of Ireland. The other plausible confounder could be a difference in coding of the STS by the cancer registries of different countries. The exact reason(s) behind this witnessed variation in rates is, however, not fully elucidated.

Trends in incidence rates in Ireland compared to other countries need to be interpreted with caution due to the significant changes in coding practices of STS over recent decades. A key example is GIST, which was likely coded as other STS types (e.g., leiomyosarcomas) before 2000. RARECARE exclude GIST from their overall STS grouping, hence, before the introduction of ICD‐O3, tumors that were actually GISTs were likely coded as leiomyosarcomas (and so included in the STS grouping) This would also affect the incidence rates over time, particularly with small overall numbers in countries of limited size.

The ASRs of Ireland were obtained for 19 years, enabling us to observe trends in the incidence of STS over time. There was an overall gradual increase in ASR over the entire period with peak incidence in 2003, this is comparable to some extent with the overall increase in incidence rates of STS worldwide most probably due to better recognition and diagnosis as well as the influence of risk factors 19. This increase in incidence was highly significant statistically. Trends in incidence rates should be interpreted with caution due to changing coding practices over time. Kaposi sarcomata are not included in the STS grouping so should not affect incidence figures.

Studies from the United Kingdom, United States and Europe 2, 4, 15, 18 also reported highest incidence of STS in extremities particularly lower limbs and buttocks, comparable to our report.

The incidence of STS in Ireland increased rapidly after the age of 60 years, with peak incidence between 75 and 79 years of age; similar to international reports 2, 4, 15, 18. Common tumor sites in different age groups were also similar to other population‐based studies 2, 4.

The age‐adjusted incidence rates of STS were higher in females compared to males in Ireland (M:F = 0.83:1). The international comparison of gender predilection is divergent. In certain reports from European countries, STS is also shown to have a higher incidence in females. The RARECARE sarcoma study reports an overall higher incidence of STS in females as compared to males in Europe. This is attributed to the higher incidence of STS in uterus and breast in females as compared to the paratesticular organs in males in these countries 4. This was true in our report as ASR among females was highest in the uterus (1.44) and this was greater than ASR among males in any single site (highest in limbs: 1.09). In contrast, few other European studies, studies from the United Kingdom and the United States report a higher incidence of STS in males 15, for example, Toro et al. (U.S.A.) 2 and a regional study based in France and Italy 17. Hence, due to inconsistencies in results from various studies, clear gender predilection of STS cannot be obtained.

Among European regions, Eastern Europe and the United Kingdom and Ireland have the lowest survival rates while Southern Europe has the highest survival rate (61%) in patients with STSs 4. United States has the highest relative 5‐year survival rates (65.3%) among the published international reports 16. The overall 5‐year relative survival rates of STSs increased between 1994–1999 and 2001–2005 from 55.1% to 57.1% in Ireland. This is statistically similar when compared to 5‐year relative survival rates in the United Kingdom (54% in 1995–2000 and 56% in 2001–2005) 15. These observations are subject to differences in coding between United Kingdom and Ireland.

Relative survival was very low in genitourinary tumors of females including uterus and highest in the STS of skin, similar to published reports 4, 15. The population based study from Europe reported lowest survival rates in STS of heart and mediastinum followed by uterine STS 4. The incidence of STS in heart and mediastinum in Ireland was very low and hence it was not possible to calculate robust survival figures.

The relative 5 year survival over 1994 to 2012 was four points higher in males as compared to females (59% in males, 55% in females) which has also been reported in the United Kingdom 15 but this discrepancy in survival among genders has not been reported by other countries 4. This could be attributed to the relatively poorer survival rates for female‐specific cancers and the almost 100% survival in well differentiated liposarcomas that are on the rise in males 15. However, comparatively poor survival rates in females were also found for peritoneal sarcomas (39%) and Ewing sarcoma of soft tissue (37.5%).

In addition, the relative survival for STS in females reduced slightly over this period while in males, an increase in Ireland. This is in contrast to the study from United Kingdom where the survival in both genders only improved over time (males: 51–55% and females: 49–52%) 15. Even though the improved relative survival in males over the years in Ireland is encouraging, the cause behind the reduction in the relative survival in females requires investigation.

These survival differences could be explained by the variation in the time and accuracy of diagnosis as well as access to appropriate treatment among different countries 4. The other reason for a lower survival rate for STS in Ireland could, in part, be the lack of the requisite number of specialized centers for STS. It is widely accepted that sarcoma care should be concentrated in specialist centers with multidisciplinary expertise and knowledge of the disease, though the effect of such a policy on hard outcomes such as survival has seldom been evaluated 4. There may be a need to increase centers delivering specialized sarcoma care in Ireland. Centralizations of sarcoma care in Ireland, implemented recently may have a positive impact on the survival. Majority of all STS where stage of tumor was recorded were already at an advanced (Stage IV) at the time of diagnosis but STS in limbs was discovered in earlier. Advanced stage of tumors at presentation could be one of the reasons for the lower relative survival of STS in Ireland 19, 20. The staging data were only complete in a low number, hence a robust association with survival is not possible to make. Overall, it is necessary to investigate more definitive explanations and make recommendations to improve survival rates in Ireland.

### Limitations

The major limitation of our study is around interpreting comparisons between local and international data as well as local data interpretation over time. Incidence and survival figures are dependent on coding practices and data collection methods. In rare tumor groups like STS, grouping and coding can get significantly complicated owing to the complex heterogenous nature of these tumors. Changing coding practice of these tumors over time significantly affects trends in incidence and survival rates. However, NCRI collects data from multiple data sources to ensure completeness of reporting. In this study, care has been taken to adhere strictly to the STS definition described by the RARECARE group, thereby allowing us to compare Irish incidence and survival rates to those reported for Europe.

## Conclusions

The incidence trends of STS in Ireland were similar to other international reports. The 5‐year survival rate of STS is lower than Southern Europe and the United States. Although survival has increased over time, it still lags behind comparable healthcare districts. The 5‐year survival rate of STS in females in Ireland has decreased over the past few years, whereas in males, it has increased significantly. Further detailed study is urgently required to elucidate these observations.

## Conflict of Interest

None declared.
